# Lost in translation: Molecular basis of reduced flower coloration in a self-pollinated monkeyflower (*Mimulus*) species

**DOI:** 10.1126/sciadv.abo1113

**Published:** 2022-09-14

**Authors:** Mei Liang, Caitlin E. Foster, Yao-Wu Yuan

**Affiliations:** ^1^ Department of Ecology and Evolutionary Biology, University of Connecticut, Storrs, CT 06269, USA.; ^2^ Institute for Systems Genomics, University of Connecticut, Storrs, CT 06269, USA.

## Abstract

Phenotypic evolution is usually attributed to changes in protein function or gene transcription. In principle, mutations that affect protein abundance through enhancing or attenuating protein translation also could be an important source for phenotypic evolution. However, these types of mutations remain largely unexplored in the studies of phenotypic variation in nature. Through fine-scale genetic mapping and functional interrogation, we identify a single nucleotide substitution in an anthocyanin-activating *R2R3-MYB* gene causing flower color variation between a pair of closely related monkeyflower (*Mimulus*) species, the hummingbird-pollinated *Mimulus cardinalis*, and self-pollinated *Mimulus parishii*. This causal mutation is located in the 5′ untranslated region and generates an upstream ATG start codon, leading to attenuated protein translation and reduced flower coloration in the self-pollinated species. Together, our results provide empirical support for the role of mutations affecting protein translation, as opposed to protein function or transcript level, in natural phenotypic variation.

## Teaser

A 5′UTR mutation of a regulator of pigment biosynthesis causes natural flower color variation by affecting protein translation.

## INTRODUCTION

Cis-regulatory mutations have been widely recognized as a major source of phenotypic evolution (*1*–*5*). In principle, cis-regulatory mutations can alter gene expression through a series of linked processes, from transcription to splicing, from mRNA stability to protein translation. However, previous studies on the role of cis-regulatory elements in phenotypic evolution have, by and large, focused on only cis-elements that control gene transcription instead of the other processes of gene expression (*6*–*13*). Recent advances in proteomics led to the revelation that, in many cases, the steady-state mRNA level is poorly correlated with protein abundance (*14*). This raises the possibility that cis-regulatory mutations affecting protein abundance, as opposed to the transcript level, may also be an important contributor to phenotypic evolution but have been neglected to date due to technical challenges in protein quantification.

**Fig. 1.**
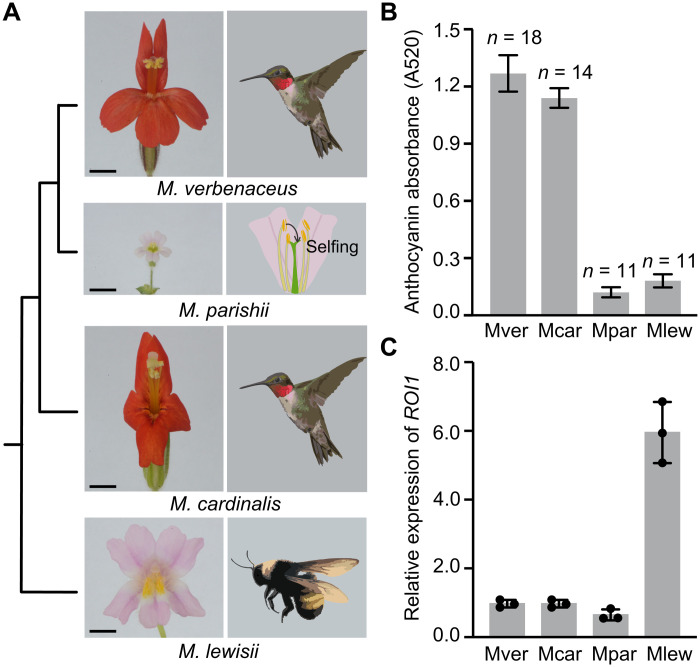
Flower color variation in the *M. lewisii* species complex. (**A**) Phylogenetic relationship among *M. lewisii*, *M. parishii*, *M cardinalis*, and *M. verbenaceus*, with all internal branches supported by 100% bootstrap values based on maximum likelihood analysis of genome-wide variants in (*23*). Scale bars, 10 mm. Pollination syndromes are shown alongside the flower images. (**B**) Relative petal lobe anthocyanin concentrations of the four species, as reflected by pigment absorbance at 520 nm. Error bars represent 1 SD from 11 to 18 flowers. (**C**) qRT-RCR of *ROI1* in the petal lobes of the four species, sampled at 1 day before flower opening. Error bars are 1 SD from three biological replicates.

A classical plant system for studying the genetic basis of phenotypic evolution, reproductive isolation, and speciation is the monkeyflower species complex (*15*–*21*), which includes the bumblebee-pollinated *Mimulus lewisii*, the hummingbird-pollinated *Mimulus cardinalis* and *Mimulus verbenaceus*, and the self-pollinated *Mimulus parishii* (Fig. 1A). The marked difference in flower color between *M. lewisii* and *M. cardinalis* has been demonstrated to play a critical role in pollinator preference in the natural habitat (*16*, *18*). The light pink color of the *M. lewisii* petal lobes (marked by “PL” in fig. S1A) is due to the low concentration of anthocyanin pigments, whereas the bright red color of *M. cardinalis* results from a combination of high concentrations of anthocyanins and carotenoids. The difference in petal lobe anthocyanin content between the two species is largely attributed to the quantitative trait locus *ROSE INTENSITY1* (*ROI1*) (*22*). *ROI1* encodes an R3-MYB repressor of the anthocyanin biosynthesis pathway and has a much higher transcript level in *M. lewisii* petal lobes than in *M. cardinalis*.

The most recent phylogenomic analysis of this species complex (Fig. 1A) (*23*) suggests that the self-pollinated *M. parishii* may have evolved low anthocyanin concentration in the petal lobes (Fig. 1B) independently of *M. lewisii*. However, the molecular basis of the reduced flower coloration in this self-pollinated species is unknown. The initial objective of this study was to determine whether the apparent parallel evolution at the phenotypic level (i.e., low anthocyanin concentration) reflects parallel evolution at the genetic level (i.e., high *ROI1* transcript level). Through genetic mapping and functional experiments, we found that the low anthocyanin content of the *M. parishii* flowers was not caused by *ROI1* but instead was caused by reduced activity of an R2R3-MYB activator of the anthocyanin biosynthesis pathway. Notably, this decreased activity is not due to coding DNA change or cis-regulatory mutations affecting gene transcription. Instead, the causal mutation is a single nucleotide substitution in the 5′ untranslated region (5′UTR) that severely decreases the translation efficiency of the *R2R3-MYB* gene in the self-pollinated species.

## RESULTS

### *ROI1* is not responsible for the pale pink color of *M. parishii* flowers

Given that the light pink color of *M. lewisii* flowers is primarily due to the high expression level of the anthocyanin repressor *ROI1* (*22*), we first suspected that *ROI1* would also be highly expressed in the pale pink *M. parishii* flowers. To test this possibility, we performed reverse transcription polymerase chain reaction (RT-PCR) of *ROI1* on the corollas of the four closely related species across multiple stages of flower development (fig. S1A). The *ROI1* transcript level gradually increased as flowers developed until 1 day before anthesis in all four species (fig. S1B). Quantitative RT-PCR (qRT-PCR) at the peak expression stage (1 day before anthesis) showed that, unexpectedly, the transcript level of *ROI1* in *M. parishii* petal lobes is comparable to the red-flowered species, ~9-fold lower than that in *M. lewisii* (Fig. 1C). This suggests that, despite the close phylogenetic relationship between the two species, the low anthocyanin concentrations in *M. parishii* and *M. lewisii* flowers are caused by different mechanisms.

### Genetic mapping identified *PELAN* as the candidate gene underlying flower color variation between *M. parishii* and *M. cardinalis*

**Fig. 2.**
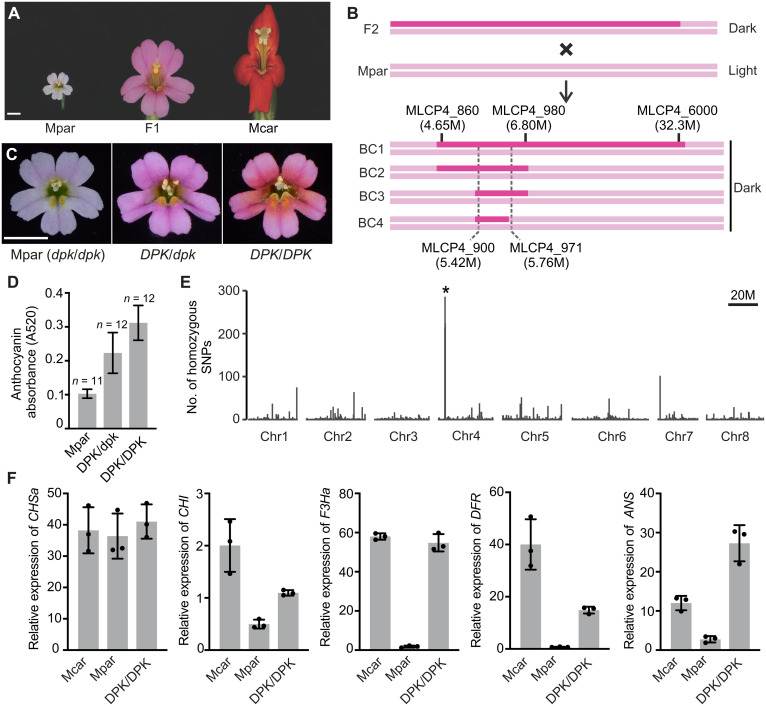
NIL construction and genetic mapping of *DPK*. (**A**) Flower images of *M. parishii* (Mpar), *M. cardinalis* (Mcar), and their F1 hybrid. Scale bar, 5 mm. (**B**) Cross-design for the NIL construction and the fine-scale mapping through a series of backcrosses. The most informative recombinant from each backcross is shown with the introgressed fragment indicated by marker positions on chromosome 4 of *M. parishii*. Flower color phenotypes (dark versus light pink) are indicated on the right. The final dark-flowered recombinant reduced the candidate interval to a 337-kb region between markers MCLP4_900 and MCLP4_971. (**C**) Flower images of *M. parishii*, the heterozygous and homozygous *DPK* NILs in the *M. parishii* background. Scale bar, 5 mm. (**D**) Relative anthocyanin concentration in the petal lobes of *M. parishii* and the *DPK* NILs, as reflected by pigment absorbance at 520 nm. Sample sizes are shown above the error bars (1 SD). (**E**) Whole genome scan of the *DPK/DPK* NIL for regions that are enriched in homozygous SNPs compared to the *M. parishii* reference genome. The peak indicated by the asterisk corresponds to the introgressed fragment from *M. cardinalis*. (**F**) qRT-PCR of early (*CHSa* and *CHI*) and late (*F3Ha*, *DFR*, and *ANS*) anthocyanin biosynthesis pathway genes [as defined in (*24*)] in the petal lobes of *M. cardinalis*, *M. parishii*, and the *DPK/DPK* NIL at the stage of 1 day before anthesis. Error bars are 1 SD from three biological replicates.

To investigate the genetic basis underlying the pale pink color of *M. parishii* flowers, we crossed *M. parishii* and *M. cardinalis*, producing F_1_ hybrids with dark pink flowers (Fig. 2A). From an F_2_ population of 325 individuals, we selected one individual that was most similar to *M. parishii* in both floral and vegetative morphology but with dark pink flowers (fig. S2) and backcrossed it to *M. parishii*. The flower color in the first-generation backcross (BC_1_) population segregated 1:1 (10:14; chi-square test, *P* = 0.414) for dark pink versus pale pink, suggesting a single causal locus. We named this locus *DARK PINK* (*DPK*) and here will refer to the recessive *M. parishii* allele as *dpk*. Initial mapping using the small BC_1_ population located *DPK* to a large interval on chromosome 4, between markers MLPC4_860 and MLPC4_6000 (Fig. 2B).

Fine-scale recombination mapping of *DPK* was carried out in concert with the construction of a near-isogenic line (NIL) in the *M. parishii* genetic background through serial backcrossing. After each backcross, one dark-flowered recombinant with the smallest introgressed fragment from *M. cardinalis* was selected for the next round. The resulting NIL after four backcrosses contained a 337-kb introgressed fragment that includes *DPK* (Fig. 2B). Selfing the heterozygous NIL (*DPK/dpk*) produced a homozygous NIL (*DPK*/*DPK*) (Fig. 2C) with even higher petal lobe anthocyanin concentration (Fig. 2D), suggesting semidominance of the *M. cardinalis* allele. Whole-genome sequencing of the NIL confirmed that the *DPK* locus was the only region introgressed from *M. cardinalis* (Fig. 2E).

The 337-kb introgressed fragment harbors 32 annotated genes (fig. S3A and table S1). One of these genes is *PETAL LOBE ANTHOCYANIN* (*PELAN*), an anthocyanin-activating *R2R3-MYB* gene previously characterized in *M. lewisii* (*24*). *PELAN* is required for anthocyanin accumulation in the petal lobes, where it activates three late anthocyanin biosynthesis pathway genes, *FLAVANONE 3-HYDROXYLASE* (*F3H*), *DIHYDROFLAVONOL 4-REDUCTASE* (*DFR*), and *ANTHOCYANIDIN SYNTHASE* (*ANS*) (*24*). If *PELAN* is *DPK*, then the *DPK* NIL should display coordinated up-regulation of *F3H*, *DFR*, and *ANS* compared to wild-type *M. parishii*. qRT-PCR experiments showed that the transcript levels of *F3H*, *DFR*, and *ANS* were all much higher in the *DPK/DPK* NIL than those in the wild-type *M. parishii*, whereas the early anthocyanin biosynthesis pathway genes *CHALCONE SYNTHASE* (*CHS*) and *CHALCONE ISOMERASE* (*CHI*) showed no or only slight difference between the NIL and the wild type (Fig. 2F). These results prompted us to consider *PELAN* as the most promising candidate gene for *DPK*.

### A single nucleotide substitution in the 5′UTR of *PELAN* is the causal mutation

**Fig. 3.**
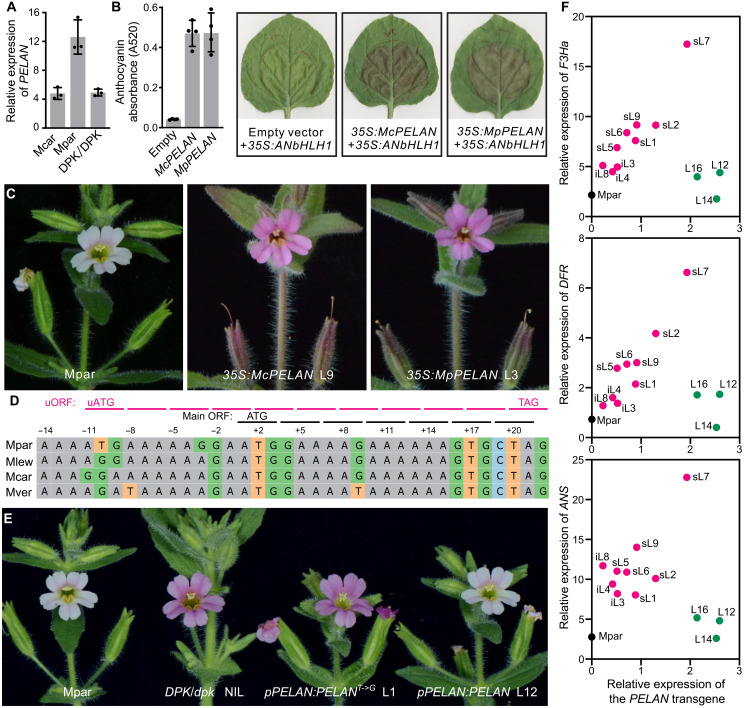
A single nucleotide substitution in the 5′UTR of *PELAN* is responsible for the variation in petal lobe anthocyanin content between *M. parishii* and *M. cardinalis*. (**A**) Relative *PELAN* transcript level, as measured by qRT-PCR of petal lobes (1 day before anthesis). Error bars are 1 SD from three biological replicates. (**B**) Transient expression assay of *MpPELAN* and *McPELAN* CDS in tobacco leaves. Error bars of anthocyanin absorbance are 1 SD from four biological replicates in one experiment. Additional experimental replicates are shown in fig. S5. (**C**) Stable overexpression of *MpPELAN* and *McPELAN* CDS in *M. parishii* (22 of the 118 *35S:McPELAN* lines and 8 of the 35 *35S:MpPELAN* lines, respectively, display the strong phenotypes). (**D**) Sequence alignment showing the normal ATG start codon (conserved among all four species) and the uATG that is unique to *M. parishii* due to a G->T substitution. The 11 codons (including the stop codon) of the uORF are highlighted in magenta. (**E**) The mutant *MpPELAN* allele, with the uATG mutated back to AGG, can restore anthocyanin accumulation in *M. parishii*, whereas the wild-type *MpPELAN* allele cannot. (**F**) Correlation between the relative transcript levels of the *PELAN* transgene and the downstream anthocyanin biosynthesis genes (*F3Ha*, *DFR*, and *ANS*), as measured by qRT-PCR. *PELAN* transgene expression was detected by a *PELAN*-specific forward primer and a vector-specific reverse primer (table S1). Each dot represents the mean of three technical replicates. The black dot indicates wild-type *M. parishii*, magenta dots indicate *pPELAN:PELAN^T->G^* lines (sL, strong lines; iL, intermediate lines), and green dots indicate *pPELAN:PELAN* lines (L12, L14, and L16 were selected because of their relatively high transgene expression levels).

To explore the molecular mechanism causing the phenotypic difference between the *DPK* NIL and the wild-type *M. parishii*, we first hypothesized that *M. parishii* has either lower *PELAN* transcript level or attenuated PELAN protein function compared to *M. cardinalis*. To evaluate the first possibility, we compared the steady-state mRNA levels of *PELAN* during corolla development in *M. parishii* and *M. cardinalis*, as well as the *DPK*/*DPK* NIL via RT-PCR. We found that transcript levels of the *M. cardinalis PELAN* allele were relatively low at early flower developmental stages and increased gradually until anthesis, in both the *M. cardinalis* (fig. S4A) and *DPK*/*DPK* NIL genetic backgrounds (fig. S4B). By contrast, transcript levels of the *M. parishii* allele were more constant during corolla development and appear higher than that of the *M. cardinalis* allele at early flower developmental stages (fig. S4C). Even at the stage of 1 day before anthesis, qRT-PCR results showed that the *PELAN* transcript level was ~2.6-fold higher in the petal lobes of *M. parishii* than in *M. cardinalis* or the *DPK*/*DPK* NIL (Fig. 3A). These observations strongly argue against the first hypothesis.

To test the second hypothesis, we aligned the predicted amino acid sequences of PELAN from the four closely related *Mimulus* species and several well-characterized PELAN homologs from other plants. Compared to the other three *Mimulus* species, *M. parishii* has two unique amino acid replacements (fig. S3B). However, these two substitutions occurred in a highly variable region of the protein, making it unlikely that PELAN function differs substantially between *M. parishii* and *M. cardinalis*. Transient expression of both the *M. parishii* and *M. cardinalis PELAN* coding sequences (CDS) in tobacco (*Nicotiana benthamiana*) leaf resulted in comparable anthocyanin accumulations (Fig. 3B and fig. S5).

To further test *PELAN* CDS function, we generated stable transgenic lines in the *M. parishii* background, expressing each CDS version driven by the constitutive CaMV 35S promoter. We found that both *35S:McPELAN* and *35S:MpPELAN* could produce dark pink flowers in the *M. parishii* background (Fig. 3C). Together, these results suggest the following: (i) The *M. parishii* and *M. cardinalis* PELAN proteins are interchangeable in function (Fig. 3, B and C); (ii) the wild-type *M. parishii* has low PELAN activity in the petal lobes, as providing that the PELAN activity by the *35S:PELAN* transgene could restore anthocyanin production (Fig. 3C); and (iii) the low PELAN activity in *M. parishii* petal lobes is not due to the insufficient steady-state mRNA level (Fig. 3A). These observations forced us to conclude that there must be mutation(s) in the *M. parishii PELAN* allele interfering with protein translation from mRNA.

Given that the 5′ and 3′UTRs often play critical roles in protein translation (*25*, *26*), we searched for mutations that are unique to *MpPELAN* by comparing the UTR sequences among the four species. A single nucleotide substitution at position −10 (relative to the predicted start codon) in the 5′UTR immediately caught our attention. This G->T substitution in *M. parishii* generates a putative out-of-frame start codon (Fig. 3D). If translated, the upstream open reading frame (uORF) adopting this upstream ATG (uATG) start codon would terminate with a stop codon at +19 and encode a peptide of only 10 amino acids (fig. S6). Therefore, we hypothesized that this G->T substitution is the causal mutation responsible for the much reduced anthocyanin concentration in *M. parishii* compared to *M. cardinalis*. To test this, we first performed transient expression assays in tobacco leaves with two contrasting constructs. The first (*35S:U377_McPELAN*) contained the wild-type *McPELAN* allele with 377-bp conserved sequence upstream of the normal ATG start codon, driven by the CaMV 35S promoter. The second construct (*35S:U377_McPELAN^G->T^*) differed from the first by only a single nucleotide, with the G at position −10 mutated to T. As expected, the mutant allele resulted in much lower anthocyanin accumulation compared to the wild-type *McPELAN* in tobacco leaves (fig. S7).

To further test this causal mutation with the native promoter and stable transgenic lines in *Mimulus*, we transformed *M. parishii* with a 3064-bp genomic fragment that contains 1761-bp sequence upstream of the normal ATG start codon and the full-length *MpPELAN* gene (including all exons and introns but excluding 3′UTR). None of the 42 resulting *pPELAN:PELAN* transgenic lines showed any obvious phenotypic change (Fig. 3E and fig. S8A). By contrast, transformation of the same *MpPELAN* genomic fragment with the T at position −10 mutated to G, which presumably represents the ancestral state (Fig. 3D), produced multiple independent *pPELAN:PELAN^T->G^* lines with dark pink (8 of 82 total lines) to intermediate (49 lines) flower colors (Fig. 3E and fig. S8B). These experiments demonstrate that *PELAN* is indeed *DPK* and that the single nucleotide substitution at position −10 is the causal mutation that reduced flower pigmentation in *M. parishii*. Notably, among the *pPELAN:PELAN^T->G^* lines with dark pink to intermediate flower colors, transcript levels of the *PELAN* transgene were positively correlated with the transcript levels of downstream anthocyanin biosynthesis genes (Fig. 3F and fig. S8). By contrast, in the pale pink *pPELAN:PELAN* lines expressing the wild-type *M. parishii* allele, transcript levels of the downstream anthocyanin biosynthesis genes were consistently low, despite that they accumulated transgene transcripts at a comparable level to the strongest *pPELAN:PELAN^T->G^* line (Fig. 3F). These observations further suggest that the role of the causal mutation does not lie in gene transcription or mRNA processing but in protein translation.

### The uATG in the 5′UTR of *MpPELAN* inhibits protein translation

**Fig. 4.**
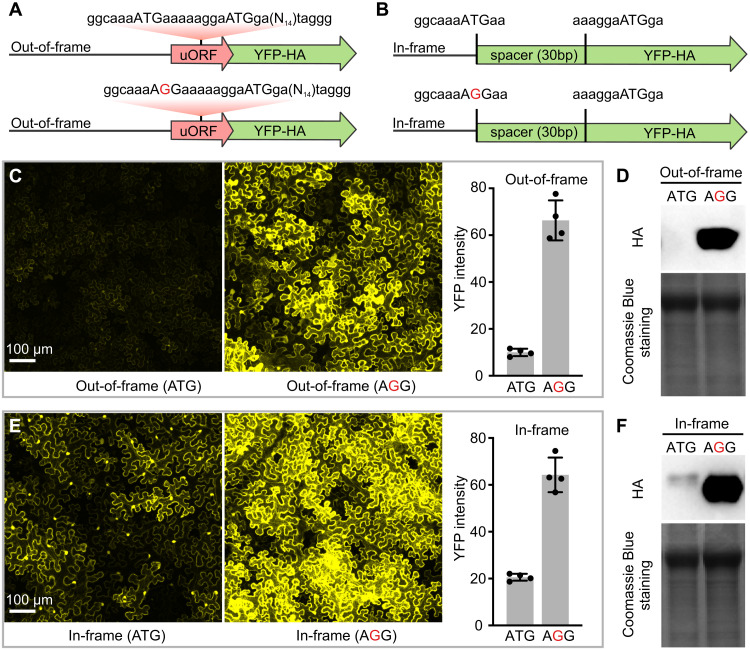
The uATG in the 5′UTR of *MpPELAN* inhibits protein translation. (**A** and **B**) Schematic illustration of the construct design. (**C**) Transient expression of *35S:uATG_OUT_ATG_YFP-HA* and *35S:AGG_OUT_ATG_YFP-HA* in tobacco leaves generates weak and strong YFP signals, respectively. Error bars of YFP intensity are 1 SD from four biological replicates. (**D**) Immunoblotting using anti-HA antibody shows that the YFP-HA chimeric protein was translated at the negligible level from the *35S:uATG_OUT_ATG_YFP-HA* construct compared to that from the *35S:AGG_OUT_ATG_YFP-HA* construct. (**E**) Transient expression of *35S:uATG_IN_ATG_YFP-HA* and *35S:AGG_IN_ATG_YFP-HA* in tobacco leaves generates weak and strong YFP signals, respectively. Error bars of YFP intensity are 1 SD from four biological replicates. (**F**) Immunoblotting using anti-HA antibody shows that both the uATG and normal ATG start codons are used for translation, as indicated by the two bands (also see fig. S10B) in the sample infiltrated with the *35S:uATG_IN_ATG_YFP-HA* construct, but the YFP-HA protein abundance is much lower in leaf tissue infiltrated with *35S:uATG_IN_ATG_YFP-HA* than that in tissue infiltrated with *35S:AGG_IN_ATG_YFP-HA*. Coomassie Blue staining in (D) and (F) shows the relative amount of total protein extracts loaded on the gel. Additional experimental replicates of these transient assays are shown in figs. S9 and S10.

To determine how this causal mutation affects protein translation, we designed four reporter constructs with the CaMV 35S promoter and a yellow fluorescent protein–hemagglutinin (YFP-HA) tag (Fig. 4, A and B), which allowed us to examine relative protein abundance via both confocal fluorescence imaging and immunoblotting. The first construct (*35S:uATG_OUT_ATG_YFP-HA*) contained the entire out-of-frame uORF of the wild-type *MpPELAN* allele (Figs. 3D and 4A). The second construct (*35S:AGG_OUT_ATG_YFP-HA*) differed from the first by only one nucleotide that changes the uATG to AGG. Transient expression of these reporter constructs in tobacco leaves through *Agrobacterium* infiltration resulted in only weak YFP signals from *35S:uATG_OUT_ATG_YFP-HA* but very strong YFP signals from *35S:AGG_OUT_ATG_YFP-HA* (Fig. 4C and fig. S9A), despite similar levels of *YFP-HA* transcripts in the infiltrated tissues (fig. S9C). Consistently, immunoblot analysis using anti-HA tag antibody hardly detected any protein products from the first construct but detected a strong band from the second (Fig. 4D and fig. S9B). These results confirmed that the presence of the uATG attenuates the translation of the normal ORF.

To verify that the uATG can be used for protein translation, we designed the third reporter construct (*35S:uATG_IN_ATG_YFP-HA*) that placed the uATG and the normal ATG in the same reading frame, separated by a 30-bp spacer, and the fourth construct (*35S:AGG_IN_ATG_YFP-HA*) that differed from the third by only one nucleotide (Fig. 4B). Transient expression of these two reporter constructs in tobacco leaves resulted in strong YFP signals from *35S:AGG_IN_ATG_YFP-HA* but much weaker signals from *35S:uATG_IN_ATG_YFP-HA* (Fig. 4E and fig. S10A), despite similar levels of *YFP-HA* transcripts in the infiltrated tissues (fig. S10C). Immunoblot analysis using anti-HA tag antibody showed that both ATG start codons were used for protein translation, as indicated by the presence of two protein products (Fig. 4F and fig. S10B). However, the proteins translated from the mRNA with both the uATG and the normal ATG start codons were much less abundant than that translated from the mRNA with only the normal start codon, further confirming that the uATG severely inhibits protein translation.

## DISCUSSION

In this study, we identified a single nucleotide substitution in an anthocyanin-activating *R2R3-MYB* gene that is responsible for the evolution of reduced flower coloration in a self-pollinated monkeyflower species. Intriguingly, this causal mutation is not located in either the coding DNA region or cis-regulatory elements that control gene transcription. Instead, it lies in the 5′UTR and generates an uATG start codon, leading to attenuated protein translation.

About 50% of mammalian transcripts (*27*, *28*) and 30 to 45% of angiosperm mRNAs (*29*) contain at least one uATG in their 5′UTRs. It has been shown that these uATGs and associated uORFs play a general repressive role in the translation of the main ORF across a broad suite of genes in a wide range of organisms (*27*, *30*). Both the gain and loss of uATGs are simple mutational events, as it takes only a single nucleotide change in many sequence contexts (e.g., AGG, ACG, ATA, and ATC). Hence, one would expect that mutations causing the gain or loss of uATGs should be a common source for phenotypic evolution in nature. However, because protein abundance is much more challenging to assess than transcript level, a causal link between mutations affecting protein translation and phenotypic variation is hard to establish, which may explain the neglect of this type of mutations in the current literature on the genetic basis of phenotypic evolution. Our results also highlight the power of functional tools in pinpointing the molecular bases of phenotypic variation. Without the ability to perform transgenic experiments, we might have ruled out *PELAN* as the causal gene of flower color variation after finding out that neither the transcript level nor the protein function explains the phenotypic difference between *M. parishii* and *M. cardinalis*.

The *M. lewisii* species complex is a good example demonstrating the importance of “going for the genes” (*31*) to understand phenotypic evolution. On the basis of detailed genetic analyses and functional characterization of *ROI1* in our previous work (*22*) and *PELAN* in the present study, we now know that the pale/light pink color of *M. lewisii* and *M. parishii* evolved independently through different genetic mechanisms. The former is due to cis-regulatory change in the promoter region of the anthocyanin repressor *ROI1* that increased its transcript level, whereas the latter is due to cis-regulatory mutation in the 5′UTR of the anthocyanin activator *PELAN* that decreased its protein abundance. Because each evolutionary transition occurred in only one species, we can confidently infer that the high expression level of *ROI1* and low protein abundance of PELAN are derived traits unique to *M. lewisii* and *M. parishii*, respectively. In other words, the common ancestor of this species complex likely had high floral anthocyanin content, as in the hummingbird-pollinated *M. cardinalis* and *M. verbenaceus*, and anthocyanin intensity was reduced independently in the bumblebee- and self-pollinated species. This interpretation is in sharp contrast to the prevailing view that floral trait evolution usually follows the direction of pollination syndrome transitions from bee to hummingbird to hawkmoth pollination [(*32*–*34*) but see (*35*)]. Recent studies of three closely related *Petunia* species revealed similar surprises in the direction of flower color evolution: Both the purple, bee-pollinated flower and the red, hummingbird-pollinated flower evolved from a white, hawkmoth-pollinated ancestor (*13*, *36*). The white-flowered species expresses a pseudogenized *R2R3-MYB* gene *AN2*, which is a homolog of *PELAN*. In the purple-flowered species, *AN2* was resurrected by a reading-frame-restoring mutation. In the red-flowered species, anthocyanin pigmentation was restored by the gain of expression of an *AN2* paralog. Without a clear understanding of the molecular mechanisms, the directions of these evolutionary transitions would not have been possible to conclusively determine based on phylogenetic relationships alone. With rapid technological advances in proteomics and more organisms becoming amenable to rigorous functional interrogation, we expect that cis-regulatory mutations altering protein abundance will be unmasked more frequently in future genetic analyses of phenotypic variation and that detailed molecular characterization of the causal genes and mutations will continue revealing the fascinating intricacy of phenotypic evolution.

## MATERIALS AND METHODS

### Plant materials and growth conditions

A highly inbred line was used for each of the four *Mimulus* species. The inbred lines for *M. lewisii* (LF10), *M. cardinalis* (CE10), and *M. verbenaceus* (MvBL) were described previously (*22*, *37*). The inbred line for *M. parishii* was generated by self-pollination and single-seed descent for >10 generations from a naturally selfing plant collected from Deep Creek near Palm Springs, CA (original seeds were provided by L. Fishman, University of Montana). All plants were grown in the University of Connecticut Ecology and Evolutionary Biology (EEB) research greenhouses, under natural light supplemented with sodium vapor lamps ensuring a 16-hour day length, with daytime temperature of ~20°C and nighttime temperature of ~15°C. Plants were fertilized three times a week.

### Anthocyanin quantification

To quantify relative anthocyanin concentration of the petal lobes of the four *Mimulus* species, anthocyanins from first-day open flowers were extracted in 100 μl of methanol with 1% (v/v) HCl. For *M. lewisii*, *M. cardinalis*, and *M. verbenaceus*, the ventral petal lobe was used for extraction; for *M. parishii*, all five petal lobes were used because of the small size of its flower. The corresponding petal lobes were scanned using an Epson WorkForce Pro WP-4530 printer, and their areas were measured using ImageJ. Anthocyanin absorbance was recorded at 520 nm and normalized by a petal lobe area to account for different petal sizes of the four species. To quantify relative anthocyanin concentration of tobacco leaf tissue after transient assays, three disks were punched from the surrounding area of each injection site with a 1.5-ml Eppendorf tube, and anthocyanins were extracted in 300 μl of methanol with 1% (v/v) HCl. Anthocyanin absorbance was subsequently recorded at 520 nm.

### NIL construction and genetic mapping

We took an NIL approach to isolate the major locus controlling petal lobe anthocyanin variation between *M. cardinalis* and *M. parishii*. From a 325-individual F_2_ population, we selected one individual most closely resembling *M. parishii* but with dark pink flowers to backcross to *M. parishii*. Initial mapping using a small BC_1_ population (24 individuals) located *DPK* to chromosome 4, between markers MLPC4_860 and MLPC4_6000 (marker information is available from http://mimubase.org/markers). A dark-flowered BC_1_ individual was used for the second round of backcrossing. From the 147-individual BC_2_ population, a dark-flowered recombinant with the smallest introgressed fragment from *M. cardinalis*, determined by genotyping multiple markers between MLPC4_860 and MLPC4_6000, was selected for the third round of backcrossing (BC_3_: 98 individuals). The same strategy was applied to the fourth round of backcrossing (BC_4_: 823 individuals). The introgressed fragment in the resulting BC_4_ NIL was shortened to 337-kb.

To confirm the high quality of the final NIL, whole-genome sequencing was conducted on a BC_4_S_1_ homozygote (*DPK*/*DPK*) individual at Novogene (https://en.novogene.com/). Approximately 24-Gb, 150-bp paired-end reads were generated using the NovaSeq PE150 platform. The short reads [National Center for Biotechnology Information Sequence Read Archive (NCBI SRA) BioProject: PRJNA790954] were mapped to the *M. parishii* genome (http://mimubase.org/FTP/Genomes/Mparg_v2.0/) using CLC Genomics Workbench 7.0 (Qiagen) and then scanned for regions enriched with homozygous SNPs to identify the introgressed DNA.

### Plasmid construction

To test the anthocyanin-activating function of the *M. parishii* and *M. cardinalis* PELAN proteins (MpPELAN and McPELAN), the full-length CDS of *MpPELAN* and *McPELAN* were separately amplified from the corresponding petal cDNAs and cloned into the pEarleyGate 202 vector (*38*), which drives the expression of the transgene with the CaMV 35S promoter. To perform the transient expression in tobacco, we also built a *35S:ANbHLH1* plasmid using the same approach, as anthocyanin production in tobacco leaves requires coexpression of the R2R3-MYB and its bHLH coactivator.

To build the *35S:U377_McPELAN* plasmid for transient assay, we amplified a 1710-bp fragment from *M. cardinalis* genomic DNA, containing 377-bp sequence upstream of the normal ATG start codon that is conserved among the four *Mimulus* species and the genic sequence of *McPELAN*. To test whether the G->T substitution at position −10 would attenuate the anthocyanin-activating function of *McPELAN*, we generated the *35S:U377_McPELAN^G->T^* plasmid by mutating the “G” at position −10 to a “T” by a PCR-based approach. To this end, the primers BP-U377McPELAN-F and atgMcPELAN-R (CTTTTCCATTCTTTTTTCATTTTTT) were used to amplify the first part of the 1710-bp fragment, and the primers atgMcPELAN-F (AAAAAATGAAAAAAGAATGGAAAAG) and BP-MlcpPELAN-cdsNSR were used to amplify the second part. These two fragments were then pooled together as template, amplified by overlap extension PCR using BP-U377McPELAN-F and BP-MlcpPELAN-cdsNSR to generate a full-length mutant *McPELAN* allele with the G-to-T mutation. The resulting wild-type *McPELAN* and mutant *McPELAN^G->T^* fragments were subsequently cloned into pEarleyGate 100 (*38*), which drives the expression of the transgene with the CaMV 35S promoter.

To test whether *PELAN* is the causal gene and whether the G->T substitution in *MpPELAN* is the causal mutation underlying the anthocyanin variation between *M. parishii* and *M. cardinalis* with stable transgenics, we amplified a 3064-bp genomic region from the *M. parishii* genomic DNA. This region contains 1761-bp sequence upstream of the normal ATG start codon and the full-length *MpPELAN* gene (including three exons and two introns but excluding 3′UTR). The resulting wild-type fragment was then used as a template to amplify a mutated variant with the T at position −10 replaced by G using PCR-directed mutagenesis as described above. Subsequently, both the mutated fragment and the wild-type version were cloned into pEarleyGate 302 (*38*), to generate the final *pPELAN:PELAN^T->G^* and *pPELAN:PELAN* plasmids that drive the expression of *MpPELAN* by its native promoter.

To test the impact of the uATG on protein translation, we designed four reporter constructs for transient assay. We first cloned a 41-bp DNA fragment of the wild-type *MpPELAN* into pEarleyGate 101 (*38*), which contains the CaMV 35S promoter and a non-ATG YFP-HA tag fused in frame at the C terminus, resulting in the *35S:uATG_OUT_ATG_YFP-HA* plasmid. This 41-bp sequence includes the entire out-of-frame uORF (33-bp), six upstream nucleotides (i.e., the Kozak context of uATG), and two downstream nucleotides (i.e., to ensure the normal ATG is in-frame with YFP-HA). The *35S:AGG_OUT_ATG_YFP-HA* plasmid was built in the same way and differs from *35S:uATG_OUT_ATG_YFP-HA* by only one nucleotide, with the uATG mutated to AGG. To analyze the two start codons in the same reading frame, we cloned a 52-bp synthetic fragment into pEarleyGate 101 (*38*), resulting in the final *35S:uATG_IN_ATG_YFP-HA* plasmid. The 52-bp sequence contains the uATG start codon and the normal ATG start codon, with their flanking nucleotides (six upstream nucleotides and two downstream nucleotides) and a 30-bp spacer. The *35S:AGG_ IN_ATG_YFP-HA* plasmid was built in the same way and differs from *35S:uATG_ IN_ATG_YFP-HA* by only one nucleotide, with the uATG mutated to AGG.

Plasmids were verified by Sanger sequencing before being transformed into *Agrobacterium tumefaciens* strain GV3101 for subsequent plant transformation in *M. parishii*, following Yuan *et al.* (*22*). All primer sequences used for plasmid construction are listed in table S2.

### Transient expression assay

For transient expression assay of anthocyanin production in tobacco (*Nicotiana benthamiana*), *Agrobacteria* with the corresponding constructs were resuspended in MES buffer containing 10 mM MES, 10 mM MgCl_2_, and 150 mM acetosyringone. The resuspended solution was then infiltrated into 5-week-old leaves using a 1-ml needleless syringe. The 35S:*McPELAN*, 35S:*MpPELAN*, *35S:U377_McPELAN*, *35S:U377_McPELAN ^G->T^*, and empty vector control with equal optical density at 600 nm (OD_600_) were each mixed with *35S:ANbHLH1* before injection. The final OD_600_ of each construct was 0.3. Anthocyanin accumulation was detected 5 days after infiltration.

For transient assay of the plasmid *35S:uATG_OUT_ATG_YFP-HA*, *35S:AGG_OUT_ATG_YFP-HA*, *35S:uATG_IN_ATG_YFP-HA*, and *35S:AGG_IN_ATG_YFP-HA*, *Agrobacteria* were resuspended in the MES buffer with OD_600_ = 0.3 and were infiltrated into tobacco leaves as described above. Z-stacks of YFP fluorescence images were obtained 4 days after infiltration using an inverted Leica SP8 confocal microscope with HC PL FLUOTAR 10×/0.30 dry objective. YFP was excited at 514 nm with laser power of 7%, and emission was collected at 525 to 600 nm with a gain value of 650 and a pinhole size of 70.7 μm. Mean intensity of YFP was measured in ImageJ.

For each transient assay, half of the leaf tissue of the infiltrated area were used for pigment analysis or confocal imaging, and the remaining half were collected in liquid nitrogen and kept in −80 freezer until further RNA or protein extraction. The relative transcript level of the transgene was assayed using semi–qRT-PCR. The *Actin* gene was used as reference. RT-PCR primers are listed in table S2. Each transient expression assay was repeated for at least three times, which produced similar results.

### RNA extraction and (q)RT-PCR

RNA was isolated using the Spectrum Plant Total RNA Kit (STRN250, Sigma-Aldrich), and cDNA was synthesized from 500 ng of total RNA with GoScript reverse transcriptase (A2791, Promega) and then diluted fourfold before PCR. (q)RT-PCR was carried out as previously described (*22*). Amplification efficiency for each primer pair was determined using critical threshold values obtained from a dilution series (1:4, 1:8, 1:32, and 1:64) of pooled cDNAs. Three biological replicates were used for all qRT-PCR. *MlUBC* was used as a reference gene to normalize expression levels following the delta-delta *C*t method.

### Protein extraction and immunoblotting

Total proteins of the tobacco leaves infiltrated with *35S:uATG_OUT_ATG_YFP-HA*, *35S:AGG_OUT_ATG_YFP-HA*, *35S:uATG_IN_ATG_YFP-HA*, and *35S:AGG_IN_ATG_YFP-HA* were extracted with the buffer containing 150 mM NaCl, 50 mM tris-HCl (pH 8.0), 5 mM EDTA (pH 8.0), 5 mM dithiothreitol, 1% (v/v) Triton X-100, 10% (v/v) glycerol, and 1% (v/v) protease inhibitor cocktail. Extracts were separated using 12% SurePAGE Bis-Tris gel (M00669, GenScript) and transferred onto polyvinylidene difluoride membrane. Membrane was immunoblotted using 1:3000 primary antibodies containing anti-HA (A01244S, GenScript) and 1:10,000 anti-mouse (ab6728, Abcam) secondary antibodies. Immunoreactive bands were detected using the ECL Western blot kit (170-5061, Bio-Rad) and captured using the ChemiDoc XRS+ gel imaging system (Bio-Rad). Coomassie Blue staining for protein was served as a protein loading control.

## Notes

View/request a protocol for this paper from *Bio-protocol*.
