# Supplementary material for: Lost in translation: Molecular basis of reduced flower coloration in a self-pollinated monkeyflower (Mimulus) species
Source: Sci Adv. 2022 Sep 14;8(37):eabo1113. doi: 10.1126/sciadv.abo1113 (PMC9473569; doi:10.1126/sciadv.abo1113)
Supplement: Supplementary file 1 — Figs. S1 to S10 Tables S1 and S2 [file sciadv.abo1113_sm.pdf]

Science Advances
AAAS

Supplementary Materials for

Lost in translation: Molecular basis of reduced flower coloration in a self-pollinated monkeyflower (*Mimulus*) species

Mei Liang, Caitlin E. Foster, Yao-Wu Yuan

Corresponding author: Mei Liang, mei.liang@uconn.edu; yaowu.yuan@uconn.edu

Sci. Adv. **8**, eabo1113 (2022)
DOI: 10.1126/sciadv.abo1113

This PDF file includes:

Figs. S1 to S10
Tables S1 and S2

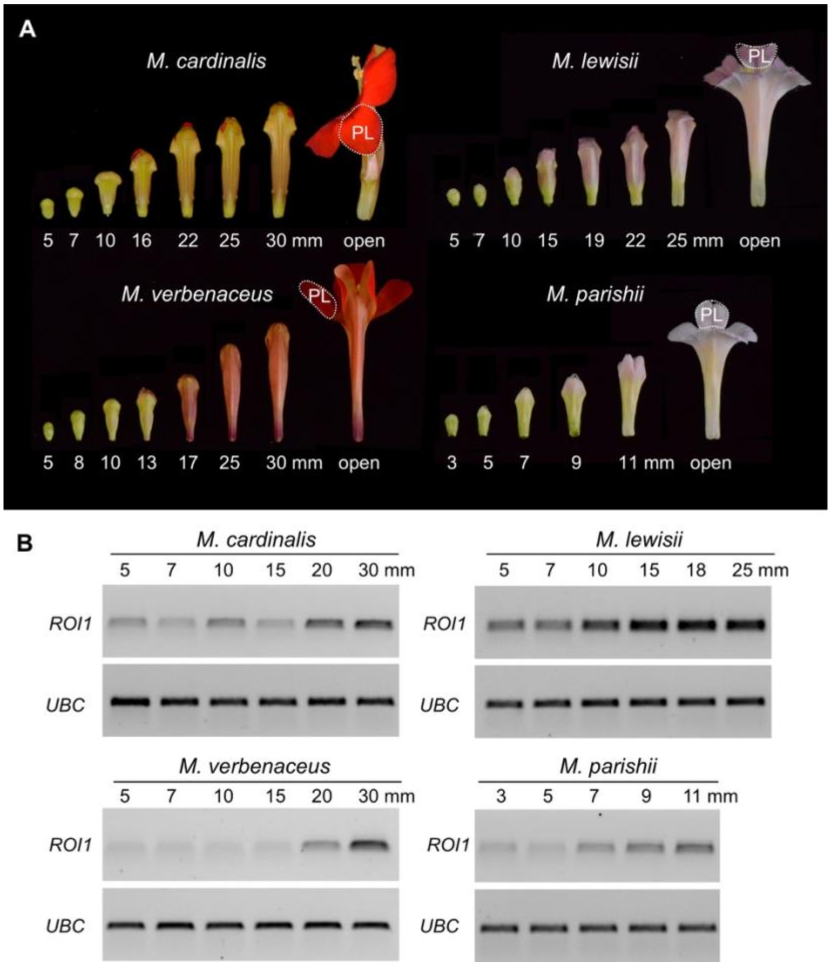

Fig. S1. Dynamics of *ROI1* expression across corolla developmental stages. (A) Corolla images of the four closely related species across multiple stages. The stage of one day before anthesis of *M. cardinalis*, *M. verbenaceus*, *M. lewisii*, and *M. parishii* corresponds to 30-mm, 30-mm, 25-mm and 11-mm, respectively. One of the petal lobes (PL) of each species is marked by the dashed line. (B) RT-PCR of *ROI1* (28 cycles) and the reference gene *UBC* (28 cycles) across multiple corolla stages.

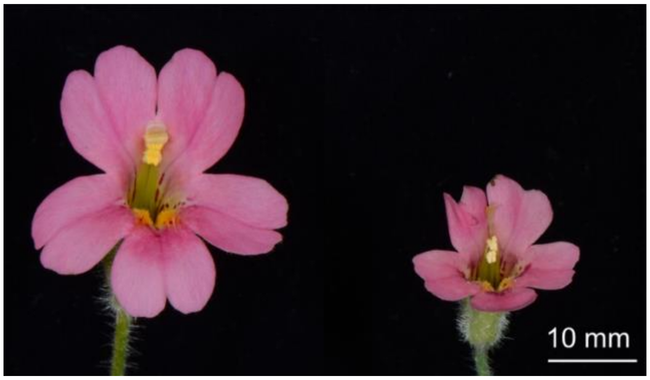

Fig. S2. Flower images of the *M. parishii* x *M. cardinalis* F1 hybrid (left) and the F2 individual (right) selected for backcrossing.

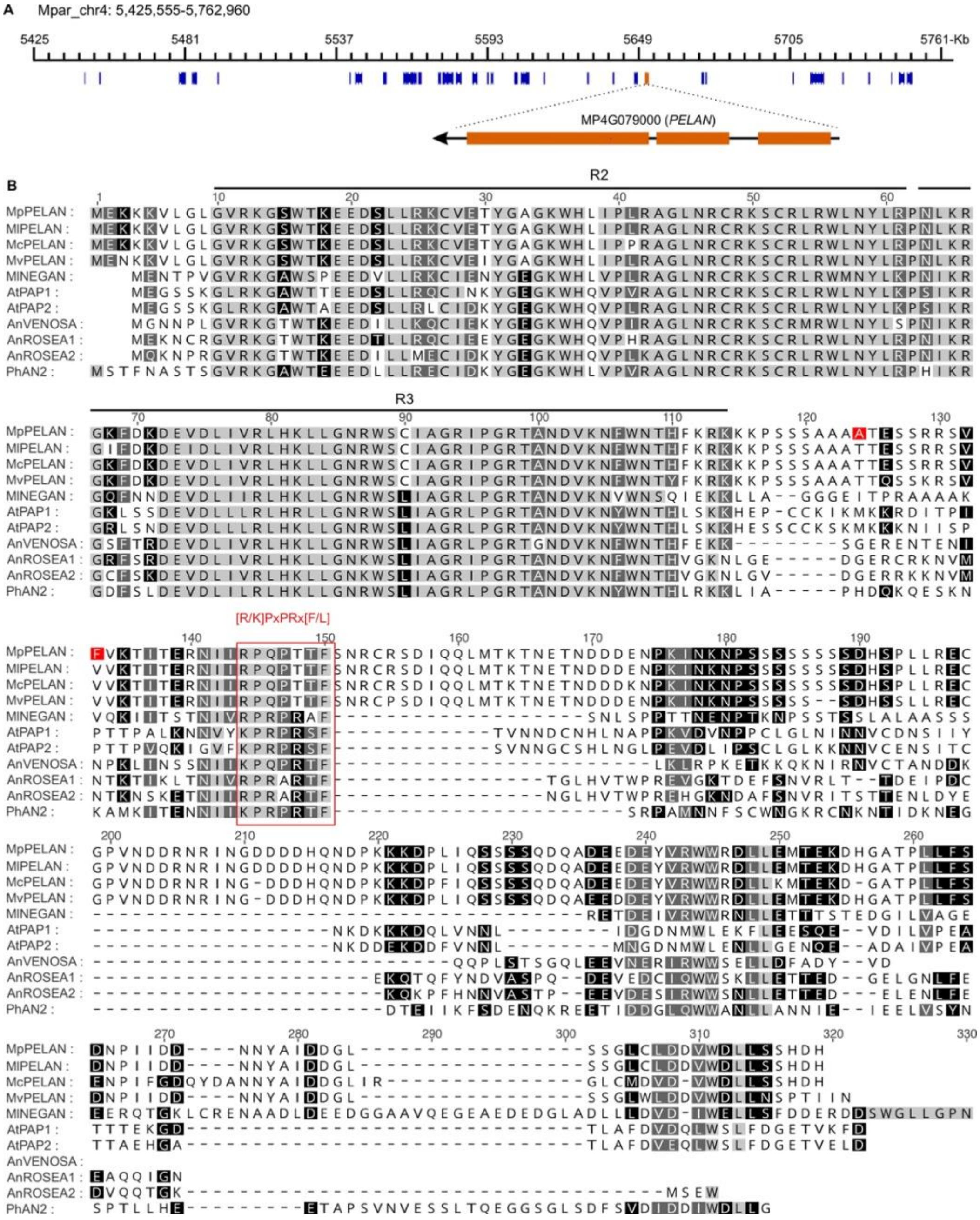

Fig. S3. Fine-scale mapping identified *PELAN* as the candidate gene. (A) The 337-Kb candidate region on chromosome 4 contains 32 annotated genes, one of which is the *R2R3-MYB* gene *PELAN* (MP4G079000), with three exons and two introns. Functional annotations of these 32 genes are listed in Table S1. (B) Alignment of the PELAN amino acid sequences from *M. parishii*, *M. lewisii*, *M. cardinalis*, *M. verbenaceus*, and their homologs from petunia (*Petunia hybrida*), snapdragon (*Antirrhinum majus*), and Arabidopsis. The R2 and R3 MYB DNA binding domains are marked by the horizontal bars above the alignment. The conserved "[R/K]PxPRx[F/L]" motif defining the subgroup 6 R2R3-MYBs that activate anthocyanin biosynthesis is indicated by the red box. Two amino acid replacements unique to *M. parishii* (highlighted by the red background) occur in the highly variable region between the R3-domain and the "[R/K]PxPRx[F/L]" motif.

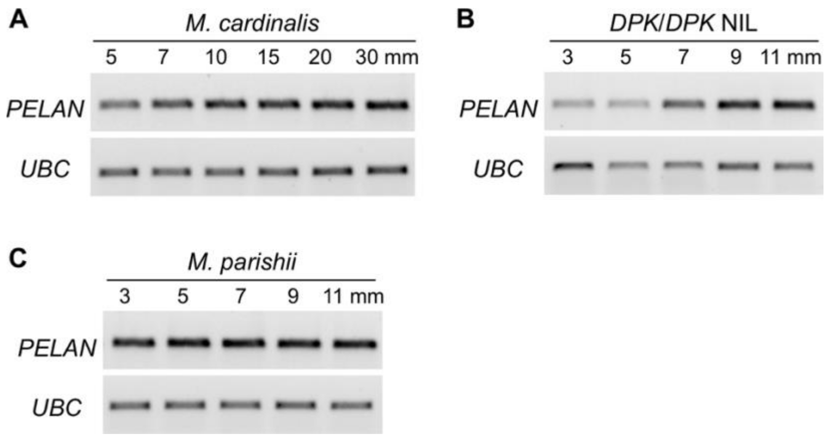

Fig. S4. Dynamics of *PELAN* expression across corolla developmental stages. RT-PCR of *PELAN* (27 cycles) and the reference gene *UBC* (28 cycles) in *M. cardinalis* (A), the *DPK/DPK* NIL (B) and *M. parishii* (C).

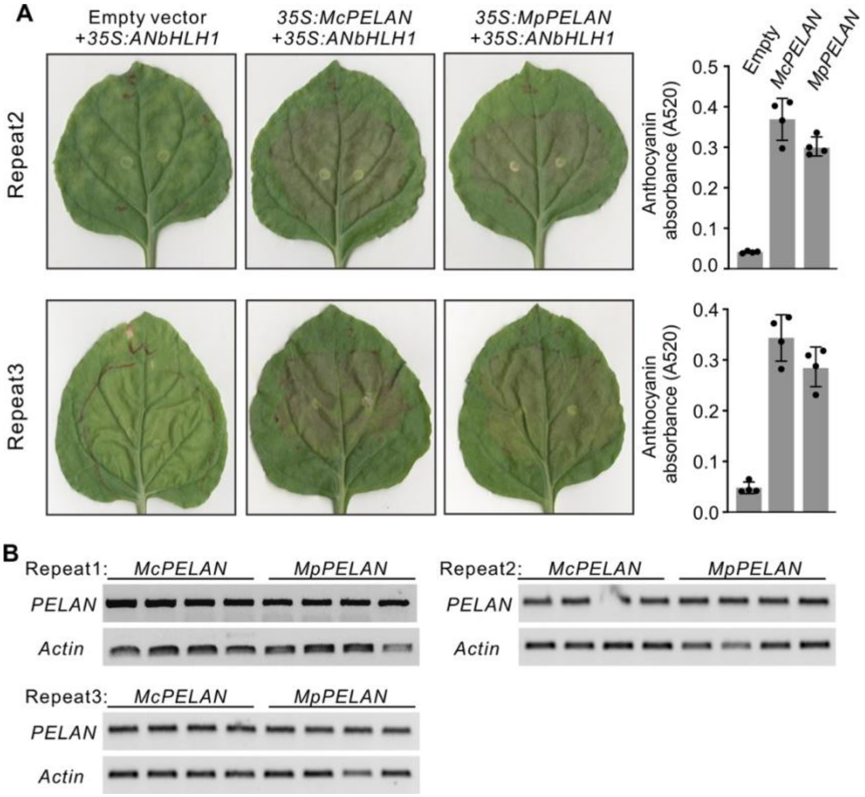

Fig. S5. Independent experimental replicates of the transient expression assay of *35S:MpPELAN* and *35S:McPELAN*. (A) Anthocyanin accumulation in tobacco leaves and relative anthocyanin concentration, as reflected by pigment absorbance at 520 nm, shown in the same fashion as Fig. 3B. (B) RT-PCR assay of the relative transcript levels of *PELAN* (28 cycles) and the reference gene *NbActin* (28 cycles) in 4 biological replicates from each experimental replicate.

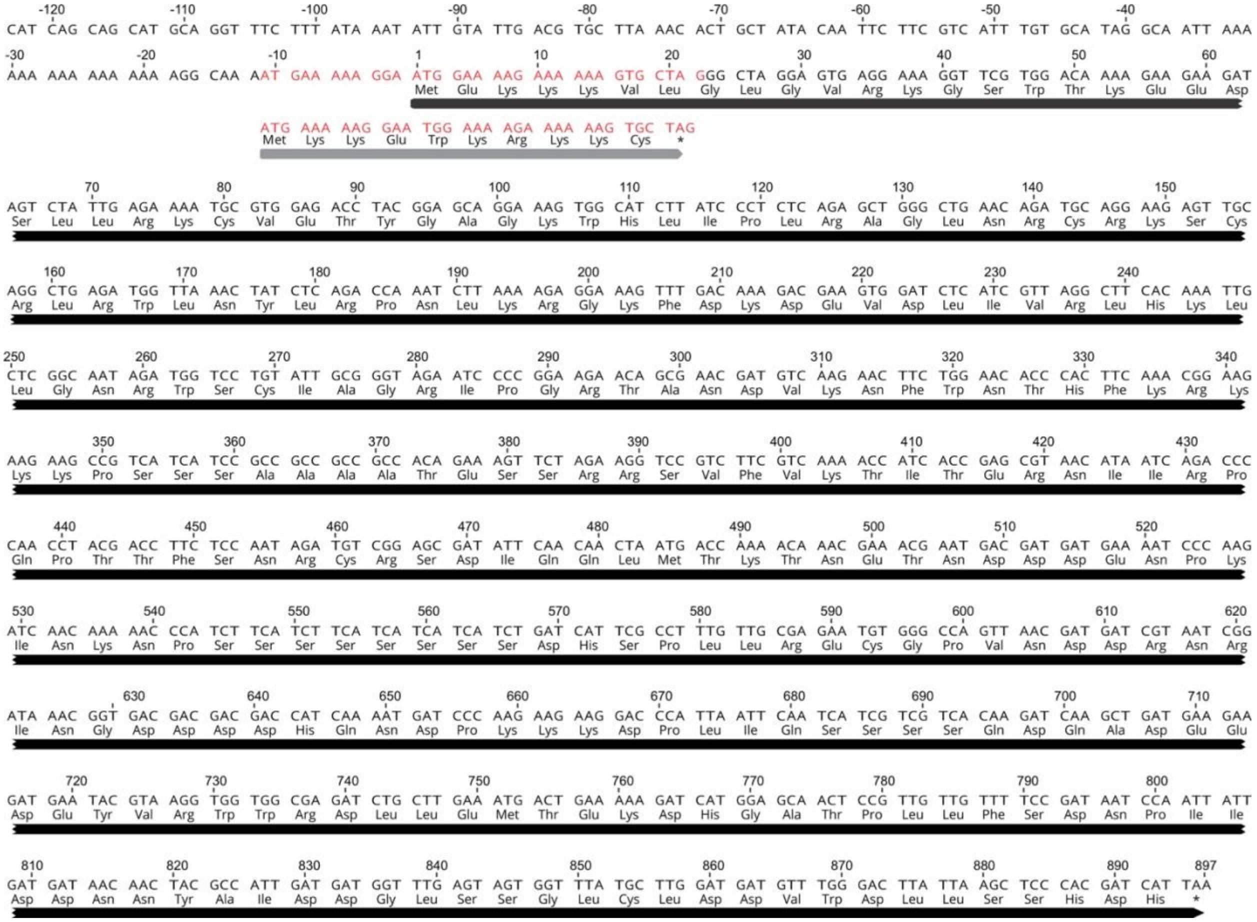

Fig. S6. Coding DNA sequence and predicted protein sequence of *MpPELAN*. The main ORF (indicated by black rectangular arrow) encodes a peptide of 291 amino acids. The uORF (indicated by grey rectangular arrow) translated from the uATG at position -10 encodes a peptide of only 10 amino acids.

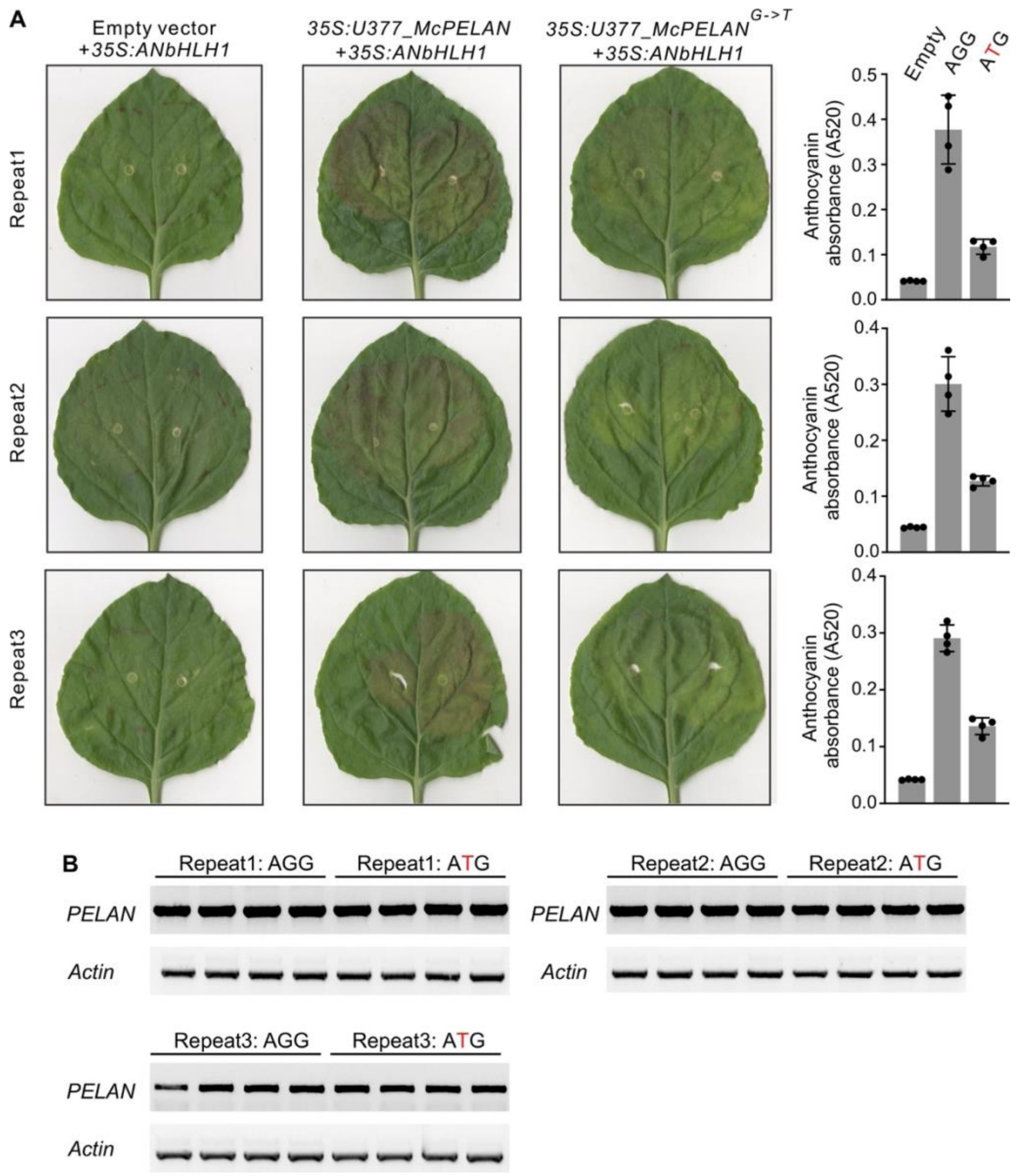

Fig. S7. Transient expression assay of *35S:U377_McPELAN* and *35S:U377_McPELAN*$^{G\text{->}T}$. Three independent experimental replicates are shown here. (A) Anthocyanin accumulation in tobacco leaves and relative anthocyanin concentration, as reflected by pigment absorbance at 520 nm. Error bars of anthocyanin absorbance are 1 SD from 4 biological replicates in each experiment. (B) RT-PCR assay of the relative transcript levels of *PELAN* (28 cycles) and the reference gene *NbActin* (28 cycles) in 4 biological replicates from each experimental replicate. "AGG" and "ATG" represent *35S:U377_McPELAN* and *35S:U377_McPELAN*$^{G\text{->}T}$, respectively.

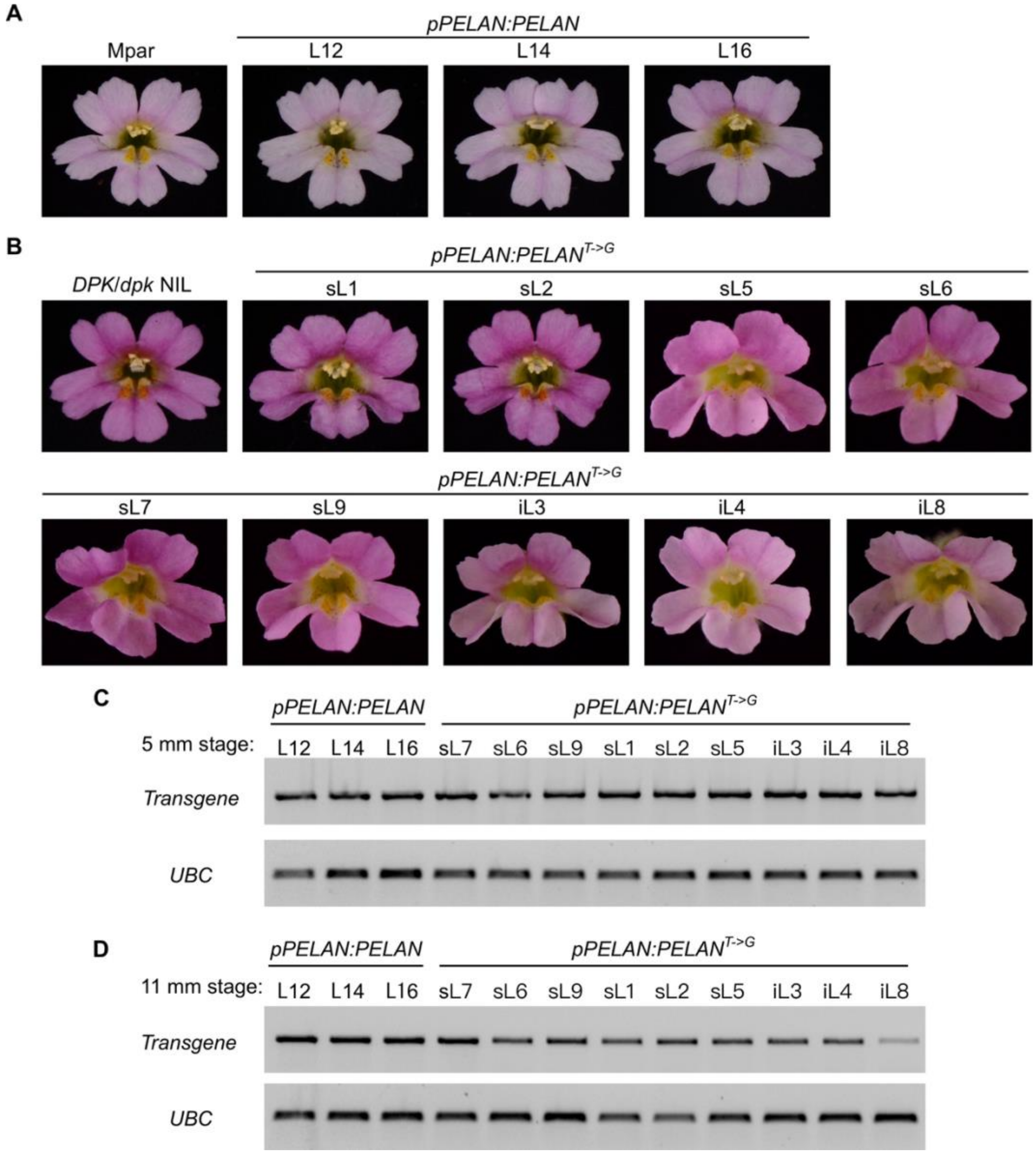

Fig. S8. Characterization of *pPELAN:PELAN* and *pPELAN:PELAN*$^{T->G}$ stable transgenic lines. (A) Flower images of three independent *pPELAN:PELAN* lines (L12, L14 and L16). (B) Flower images of six strong *pPELAN:PELAN*$^{T->G}$ lines (sL1, sL2, sL5, sL6, sL7, and sL9) and three intermediate *pPELAN:PELAN*$^{T->G}$ lines (iL3, iL4, and iL8). (C and D) RT-PCR of the *PELAN* transgene using one *PELAN*-specific primer and one vector-specific primer suggests that the pale pink color of the *pPELAN:PELAN* lines is not due to low transcript levels of the transgene. *UBC* was used as the reference gene.

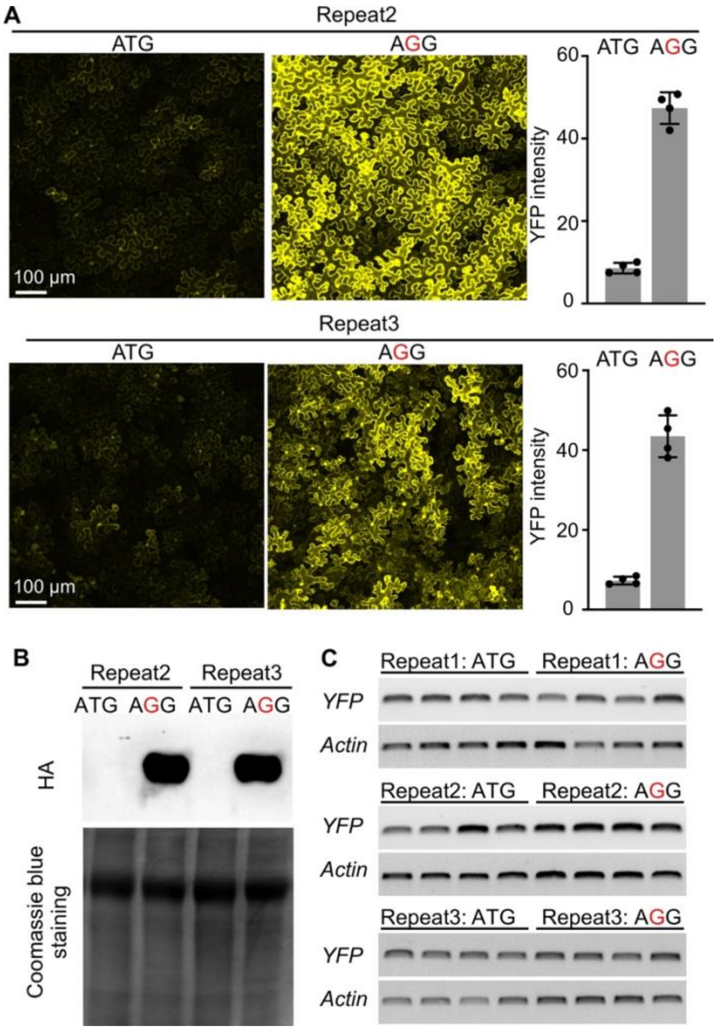

Fig. S9. Independent experimental replicates of transient expression assay of *35S:uATG_OUT_ATG_YFP-HA* and *35S:AGG_OUT_ATG_YFP-HA*. (A) and (B) are shown in the same fashion as Fig. 4C and Fig. 4D, respectively. (C) RT-PCR assay of the relative transcript levels of *YFP* (28 cycles) and the reference gene *NbActin* (28 cycles) in 4 biological replicates from each experimental replicate. "AGG" and "ATG" represent *35S:uATG_OUT_ATG_YFP-HA* and *35S:AGG_OUT_ATG_YFP-HA*, respectively.

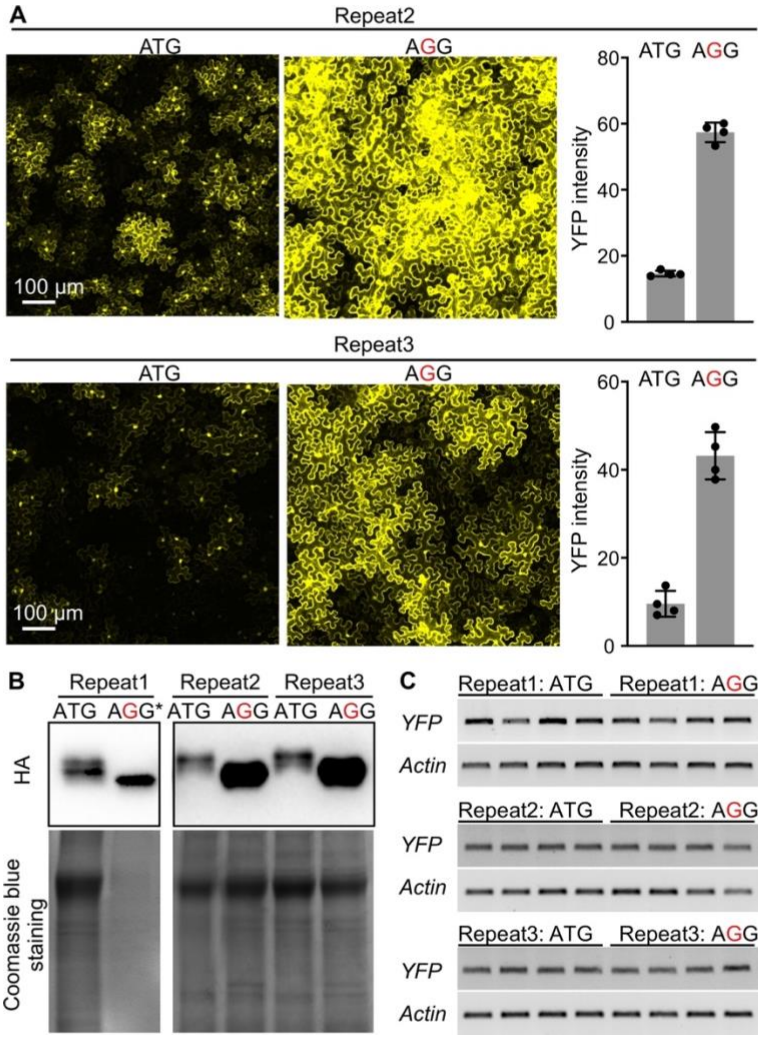

Fig. S10. Independent experimental replicates of transient expression assay of *35S:uATG_IN_ATG_YFP-HA* and *35S:AGG_IN_ATG_YFP-HA*. (A) and (B) are shown in the same fashion as Fig. 4E and Fig. 4F, respectively. Note that in the immunoblot repeat 1, the "AGG" sample (*35S:AGG_IN_ATG_YFP-HA*), as indicated by the asterisk, was diluted 10-fold from the sample shown in Fig. 4F. (C) RT-PCR assay of the relative transcript levels of *YFP* (28 cycles) and the reference gene *NbActin* (28 cycles) in 4 biological replicates from each experimental replicate. "AGG" and "ATG" represent *35S:uATG_IN_ATG_YFP-HA* and *35S:AGG_IN_ATG_YFP-HA*, respectively.

Table S1. List of annotated genes in the 337-Kb introgressed fragment.

No.	Gene_ID	*Arabidopsis* homolog	Functional annotation
1	MP4G076800	no hit	Unknown function
2	MP4G076900	AT3G52220.1	Leukocyte immunoglobulin-like receptor family A protein
3	MP4G077000	AT5G02500.1	Encodes a member of heat shock protein 70 family
4	MP4G077100	no hit	Unknown function
5	MP4G077200	no hit	Unknown function
6	MP4G077300	AT1G66480.1	Involved in chloroplast avoidance movement under intermediate and high light intensities
7	MP4G077400	AT1G22640.1	Subgroup 4 R2R3-MYB transcription factor that represses phenylpropanoid biosynthesis gene expression
8	MP4G077500	AT3G61730.1	Encodes a nuclear localized F-box protein that is involved in tapetal layer degeneration and pollen development
9	MP4G077600	AT5G37850.4	Encodes a pyridoxal kinase required for root hair development.
10	MP4G077700	AT5G17460.5	Glutamyl-tRNA (Gln) amidotransferase subunit C
11	MP4G077800	AT1G71050.1	Heavy metal transport/detoxification superfamily protein
12	MP4G077900	AT1G66520.1	Formyltransferase
13	MP4G078000	AT5G37930.1	Protein with RING/U-box and TRAF-like domain
14	MP4G078100	AT5G37930.1	Protein with RING/U-box and TRAF-like domain
15	MP4G078200	AT1G15010.1	Mediator of RNA polymerase II transcription subunit
16	MP4G078300	no hit	Unknown function
17	MP4G078400	AT1G71090.1	Auxin efflux carrier family protein
18	MP4G078500	AT2G01275.4	RING/FYVE/PHD zinc finger superfamily protein
19	MP4G078600	AT2G01300.1	Mediator of RNA polymerase II transcription subunit
20	MP4G078700	AT3G02560.3	Ribosomal protein S7e family protein
21	MP4G078800	no hit	Unknown function
22	MP4G078900	AT5G49330.1	Subgroup 7 R2R3-MYB transcription factor involved in regulation of flavonol biosynthesis
23	**MP4G079000 (PELAN)**	**AT1G66370.1**	**Subgroup 6 R2R3-MYB transcription factor involved in regulation of anthocyanin biosynthesis. Affects the expression of enzymes involved in later steps of anthocyanin biosynthesis.**
24	MP4G079100	no hit	Unknown function
25	MP4G079200	no hit	Unknown function
26	MP4G079300	no hit	Unknown function
27	MP4G079400	AT1G66680.1	Unknown function
28	MP4G079500	no hit	Unknown function
29	MP4G079600	AT3G03280.1	PADRE protein up-regulated after infection by *S. sclerotiorum*.
30	MP4G079700	no hit	Unknown function
31	MP4G079800	AT1G15125.1	S-adenosyl-L-methionine-dependent methyltransferases superfamily protein
32	MP4G079900	AT5G66090.1	Cell wall integrity/stress response component

Table S2. Primers used in this study.

Primer	Sequence (5'-3')	Utility
BPMlcp_cdsPELAN_F	GGGGACAAGTTTGTACAAAAAAGCAGGCTGCATGGAAAAGAAAAAAGTGCTAGGG	Plasmid construction (*35S:McPELAN*; *35S:MpPELAN*)
BPMlcp_cdsPELAN_R	GGGGACCACTTTGTACAAGAAAGCTGGGTCTTAATGATCGTGGGAGCTTAATAAGTC	
BP-U377McPELAN-F	GGGGACAAGTTTGTACAAAAAAGCAGGCTTCCTTTTTTTTTTTAATATGAGGGGCCCCTG	Plasmid construction (*35S:U377_McPELA*; *35S:U377_McPELA*$^{T->G}$)
atgMcPELAN-R	CTTTTCCATTCTTTTTTCATTTTTT	
atgMcPELAN-F	AAAAAATGAAAAAAGAATGGAAAAG	
BP-MlcpPELAN-cdsNSR	GGGGACCACTTTGTACAAGAAAGCTGGGTCATGATCGTGGGAGCTTAATAAGTC	
BP-proMcPELAN-F	GGGGACAAGTTTGTACAAAAAAGCAGGCTTCCAATTTTAAGCAGGCAAATAGCAG	Plasmid construction (*pPELAN:PELAN*; *pPELAN:PELAN*$^{T->G}$)
aggMpPELAN-R	CTTTTCCATTCCTTTTTCCTTTTGC	
aggMpPELAN-F	GGCAAAAGGAAAAAGGAATGGAAAAG	
BP-MlcpPELAN-cdsNSR	GGGGACCACTTTGTACAAGAAAGCTGGGTCATGATCGTGGGAGCTTAATAAGTC	
BP-outframe-uATG-F	GGGGACAAGTTTGTACAAAAAAGCAGGCTGCGGCAAAATGAAAAAGGAATGGAAAAG	Plasmid construction (*35S:uATG_OUT_ATG_YFP:HA*; *35S:uAGG_OUT_ATG_YFP:HA*)
BP-outframe-uAGG-F	GGGGACAAGTTTGTACAAAAAAGCAGGCTGCGGCAAAAGGAAAAAGGAATGGAAAAG	
BP-outframe-R	GGGGACCACTTTGTACAAGAAAGCTGGGTCCCCTAGCACTTTTTTCTTTTCCATTCC	
MLCPV_CHSa_RTF	GAGTACGGAAACATGTCGAGC	(q)RT-PCR of endogenous genes in *Mimulus*
MLCPV_CHSa_RTR	ACGACGTTGCTAACAACTGGCT	
MLCPV_CHI_RTF	TGGGAGCTCCATCCTCTTCAC	
MLCPV_CHI_RTR	CGATAATCTTGCAGCCAAACTC	
MLCPV_F3Ha_RTF	AACCCGATCTCACACTAGGCGTC	
MLCPV_F3Ha_RTR	GGTTGCTATCGATAATCTGCTGTAG	
MLCPV_DFR_RTF	TCGAGGATCCCACAGCACAAGGA	
MLCPV_DFR_RTR	TGGCTTCTCTAAACATGTCCTCCA	
MLCPV_ANS_RTF	GGGAGGACTACTTCTTCCACTG	
MLCPV_ANS_RTR	CCYAGTCCGGTTGATAGGATTG	
MLCPV_PELAN_RTF	CGAGCGTAACATAATCAGACCC	
MLCPV_PELAN_RTR	CCCACATTCTCGCAACAAAG	
MLCPV_ROI1_RTF	GGATGTATAACCTGGTTGGCG	
MLCPV_ROI1_RTR	CGATAACCAGTATTTCTTGATTTGG	
Adapter_attB1	GGGGACAAGTTTGTACAAAAAAGCAGGCT	RT-PCR of *YFP* transgene
BP-EYFP-R	TCACTTGTACAGCTCGTCCATGC	
MLCPV_PELAN_RTF	CGAGCGTAACATAATCAGACCC	RT-PCR of *PELAN* transgene
Adapter_attB2	GGGGACCACTTTGTACAAGAAAGCTGGGT	
MLCPV_UBC	GGCTTGGACTCTGCAGTCTGT	

MLCPV_UBC	TCTTCGGCATGGCAGCAAGTC	Reference gene for (q)RT-PCR in *Mimulus*
NbACTIN_F	CTGAGAGATTCCGCTGC	Reference gene for RT-PCR in *Nicotiana benthamiana*
NbACTIN_R	GAGGACAATGTTTCCGTAC	

Note: Sequences highlighted in red are the adapter sequences necessary for Gateway cloning. The mutated nucleotide is highlighted in blue.
